# “Trick or treat”: the influence of incentives on developmental changes in feedback-based learning

**DOI:** 10.3389/fpsyg.2014.00968

**Published:** 2014-09-09

**Authors:** Kerstin Unger, Berit Greulich, Jutta Kray

**Affiliations:** ^1^Developmental Cognitive Neuroscience Lab/Cognitive Neuroscience of Cognitive Control and Memory Lab, Department of Cognitive, Linguistic, and Psychological Sciences, Brown UniversityProvidence, RI, USA; ^2^Development of Language, Learning, and Action, Department of Psychology, Saarland UniversitySaarbrücken, Germany

**Keywords:** adolescence, childhood, incentives, performance monitoring, feedback-based learning, Ne/ERN, Pe

## Abstract

Developmental researchers have suggested that adolescents are characterized by stronger reward sensitivity than both children and younger adults. However, at this point, little is known about the extent to which developmental differences in incentive processing influence feedback-based learning. In this study, we applied an incentivized reinforcement learning task, in which errors resulted in losing money (loss condition), failure to gain money (gain condition), or neither (no-incentive condition). Children (10–11 years), younger adolescents (13–14 years), and older adolescents (15–17 years) performed this task while event-related potentials (ERPs) were recorded. We focused our analyses on two ERP correlates of error processing, the error negativity (Ne/ERN) and the error positivity (Pe) that are thought to reflect a rapid preconscious performance monitoring mechanism (Ne/ERN) and conscious detection and/or evaluation of response errors (Pe). Behaviorally, participants in all age groups responded more quickly and accurately to stimuli in gain and loss conditions than to those in the no-incentive condition. The performance data thus did not support the idea that incentives generally have a greater behavioral impact in adolescents than in children. While the Ne/ERN was not modulated by the incentive manipulation, both children and adolescents showed a larger Pe to errors in the gain condition compared to loss and no-incentive conditions. This is in contrast to results from adult studies, in which the Ne/ERN but not the Pe was enhanced for high-value errors, raising the possibility that motivational influences on performance monitoring might be reflected in the activity of separable neural systems in children and adolescents vs. adults. In contrast to the idea of higher reward/incentive sensitivity in adolescents, our findings suggest that incentives have similar effects on feedback-based learning from late childhood into late adolescence with no changes in preferences for “trick over treat.”

## Introduction

Adolescence has often been characterized as a period of increased reward-seeking, risk-taking and impulsive behaviors (e.g., Casey et al., 2010; Somerville and Casey, 2010). A number of influential neurodevelopmental theories share the basic notion that this unique behavioral pattern reflects a relative imbalance in the maturation of the neural systems underlying (i) emotional and incentive-driven behavior, including subcortical structures such as the amygdala and the striatum, and (ii) cognitive and emotional control, including frontal regions such as the anterior cingulate cortex (ACC) and the dorsolateral prefrontal cortex (Geier and Luna, 2009; Casey et al., 2010). Specifically, these models posit that the earlier maturation of subcortical systems can lead to a dominance of these structures over prefrontal control systems in guiding behavior, especially in situations involving salient motivational and/or social-affective cues. In contrast, prefrontal-subcortical interactions are more balanced in both children and young adults, due to a global immaturity (children) and maturity (adults) of the underlying neural circuitry. Accordingly, motivational cues like rewards are presumed to have a higher positive or negative impact on cognitive control in adolescence than at earlier or later stages of development.

In line with this view, numerous studies found that reward-related processing has a greater influence on decision-making in adolescents than in children or adults (e.g., Galvan et al., 2006; Cauffman et al., 2010; Somerville et al., 2010). While most of these studies focused on maladaptive effects of adolescents' hypersensitivity to incentives, it has recently been pointed out that pubertal changes in affective and social processing may also be associated with adaptive advantages. In particular, adolescents are thought to be biologically prepared to rapidly adjust to changing environmental conditions and hence should show a greater flexibility in the recruitment of cognitive control mechanisms to support motivational learning (cf. Crone and Dahl, 2012). Direct empirical tests of this proposal are scarce thus far. Using a reversal learning task, Van der Schaaf et al. (2011) demonstrated that adolescents are indeed better able to change their responses after unexpected rewarding and punishing outcomes compared to both children and adults. Furthermore, a developmental neuroimaging study of reinforcement learning indicated that adolescents show exaggerated striatal responses to reward prediction errors, i.e., discrepancies between expected and actually obtained outcomes (Cohen et al., 2010). Although Cohen et al. (2010) did not find age differences in overall learning performance, adolescents responded more quickly to feedback stimuli indicating large reward compared to those associated with small reward.

In the present study, we sought to expand on previous research on interactions between motivational context and learning mechanisms across adolescence by examining the impact of appetitive and aversive motivational cues on error processing and error-related performance adjustments. We used the high temporal resolution of an event-related potentials (ERPs) to track earlier and later stages of error processing as reflected in two ERP correlates of performance monitoring, the error negativity (Ne; Falkenstein et al., 1990) or error-related negativity (ERN; Gehring et al., 1993) and the error positivity (Pe; Falkenstein et al., 1990).

The Ne is a fronto-centrally distributed negative deflection that peaks ~30-100 ms after a participant's erroneous response. It is typically followed by the Pe, a slow positive wave that reaches its maximum between 200 and 400 ms after response-onset and exhibits a centroparietal scalp distribution (Falkenstein et al., 1990). While the Pe has been associated with deliberate, slower error evaluation processes, such as conscious error recognition and appraisal of the motivational significance of an error (Overbeek et al., 2005; Ridderinkhof et al., 2009; Steinhauser and Yeung, 2010), the Ne is thought to be a neural manifestation of a rapid internal response evaluation mechanism. More specifically, the Ne has been proposed to reflect the activity of a generic prefrontal performance monitoring system and to track learning-related changes in the evaluation and utilization of information about performance outcomes (Holroyd and Coles, 2002). Consistent with this notion, previous findings suggested a link between the Ne and error-induced behavioral adaptation during reinforcement learning (e.g., Frank et al., 2005; Gründler et al., 2009; Unger et al., 2012). Moreover, there is substantial evidence for motivational and affective influences on the Ne in adults (for a review, see Gehring et al., 2012). In particular, the Ne has been shown to be sensitive to the motivational value of an error (e.g., Gehring et al., 1993; Hajcak et al., 2005; Wiswede et al., 2009; Unger et al., 2012).

Developmental studies on error processing indicated that the Ne increases until mid to late adolescence (e.g., Davies et al., 2004; Ladouceur et al., 2007; for a recent review, see Ferdinand and Kray, 2014). Although some studies showed that a reliable Ne can be elicited in children as young as 5–7 years of age when using a simple Go-NoGo paradigm (e.g., Torpey et al., 2009), this component does not seem to develop until later ages for more complex tasks (e.g., Davies et al., 2004; Ladouceur et al., 2004). Eppinger et al. (2009) used a reinforcement learning paradigm to investigate age differences in error processing and found comparable accuracy rates and Ne amplitudes for children and adults in the easiest learning condition (valid feedback), while performance and Ne were increased in adults compared to children when the task was more difficult (invalid feedback). In addition, Eppinger et al. (2009) observed a larger Pe in children than adults, whereas other studies did not find age-related changes in this component (Davies et al., 2004; Ladouceur et al., 2004). The divergent findings may reflect differences in the paradigms used across studies (reinforcement learning vs. Eriksen Flanker task). Ladouceur et al. (2007), however, also reported an increase in Pe amplitude in late adolescents compared to adults, using a flanker task. This suggests that the neural processes involved in the generation of the Pe mature relatively early in development and amplitude modulations reflect age differences in error awareness or motivational significance of errors.

Available evidence regarding developmental changes of motivational influences on error processing as reflected in Ne and Pe is mixed. For instance, Kim et al. (2005) reported an increase in Ne when children performed a task while being observed by a peer compared to performing the task alone. Torpey et al. (2009), in contrast, failed to obtain significant differences in children's Ne and Pe amplitudes for high- vs. low-value errors. So far, only little is known on how motivational salience affects electrophysiological correlates of error processing and error-related performance adjustment across adolescence (cf. Ferdinand and Kray, 2014). Some insight has been gained from developmental research on the feedback-related negativity (FRN), an ERP component that has been hypothesized to reflect a rapid evaluation of the significance or value of external feedback stimuli and is thought to be functionally related to the Ne (Holroyd and Coles, 2002). Interestingly, the findings of these studies suggested that the neural system underlying the FRN differentiates less efficiently between good and bad events in adolescents compared to adults, for both small and large outcomes (e.g., Hämmerer et al., 2011; Zottoli and Grose-Fifer, 2012). The present investigation addressed the question of how motivational value affects internal rather than external indicators of performance errors. We applied an incentivized reinforcement learning paradigm in a sample of children and adolescents covering the age ranges of 10–11 years, 13–14 years, and 15–17 years. Incentive value was manipulated on a trial-to-trial basis in three different conditions: errors resulted in monetary loss (loss condition), failure to gain money (gain condition), or neither (no-incentive condition). On the basis of the theoretical considerations and previous findings outlined above, we expected adolescents to show better learning performance and larger Ne and/or Pe amplitudes in gain and loss conditions compared to the no-incentive condition, whereas incentive-related differences in behavioral and ERP measures should be less pronounced in children.

## Materials and methods

### Participants

A total of 64 children and adolescents participated in the study. Data from four participants (2 children, 2 adolescents) were excluded from analyses due to excessive artifacts in the EEG data. One child did not finish the session. The final sample thus included 59 participants from three age groups: 19 children (10–11 years, mean age = 11.02 years, 9 females), 20 mid-adolescents (13–14 years, mean age = 14.20 years, 10 females), and 20 late adolescents (15–17 years, mean age = 16.85 years, 10 females). The age ranges were chosen to (a) reflect the development of performance monitoring and reinforcement learning from preadolescence to late adolescence (e.g., Galvan et al., 2006; Cauffman et al., 2010), (b) to cover the age period during which sensitivity to incentives has been shown to peak (e.g., Somerville and Casey, 2010), and (c) to be comparable to previous developmental studies using similar paradigms (e.g., Eppinger et al., 2009; Hämmerer et al., 2011). Participants were consented in accordance with the protocols approved by the local ethics committee of Saarland University and were paid 8 Euro per hour for participation. According to self-report, all had normal or corrected-to-normal vision, no history of neurological or psychiatric illness and did not use psychoactive medication or drugs. Five participants (1 child, 3 mid-adolescents, 1 late adolescent) were left-handed, all other participants were right-handed (Oldfield Questionnaire; Oldfield, 1971). The majority of children (*N* = 16), mid-adolescents (*N* = 20) and late adolescents (*N* = 16) were attending college-preparatory high school. The parents of children had an average 16.63 (*SD* = 4.52) years of education, the parents of younger and older adolescents had an average of 16.40 (*SD* = 2.58) and 15.39 (*SD* = 2.87) years of education, respectively.

### Stimuli and task

On each trial of the reinforcement learning task, participants saw a colored image of an object (Snodgrass and Vanderwart, 1980) and chose to press one of two response keys with the left and right index finger, respectively. Feedback was presented after each choice in the form of either a happy smiley (correct response) or a sad smiley (incorrect response). Stimuli were assigned to one of three incentive conditions (gain, loss, and no-incentive condition). Each imperative stimulus was preceded by a cue that indicated the incentive value of the upcoming target. The gain cue informed participants that they would win 37 euro cents if they responded correctly but 0 euro cents if they responded incorrectly or missed the response deadline (see Trial Procedure). Conversely, the loss cue indicated that participants would lose 0 euro cents if they responded correctly but 37 euro cents if the response was incorrect or too slow. On no-incentive trials, there was no chance to gain or lose money. The outcome of each trial was indicated by “+37,” “+00,” “−00,” or “−37” signs shown together with the corresponding smiley on the feedback screen. At the end of the experiment, all participants received a performance-dependent monetary bonus (ranging between 5 and 10 Euros). In order to make the learning task more child-friendly, we constructed a cover story involving creatures living in a magic forest that have been transformed into different objects by a wizard. Participants were told that they have two magic wands (the two response keys) and should find out which one can be successfully used to free a given creature from the spell. They were further told that some creatures will reward successful retransformation with a monetary gift (gain condition), while others punish unsuccessful retransformations by taking away money (loss condition) or do neither (no-incentive condition).

### Trial procedure

**Figure 1:**
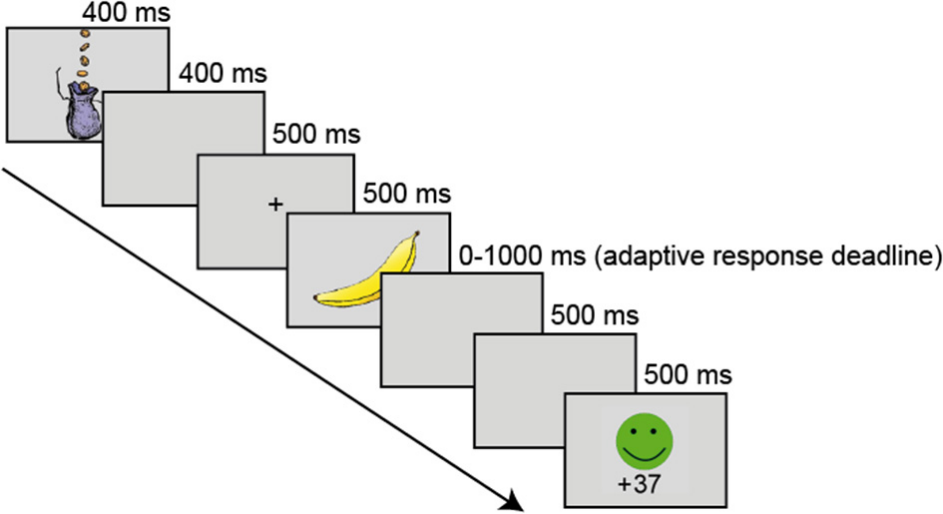
**Schematic overview of the trial procedure**.

Each trial started with the incentive cue appearing in the center of the screen for 400 ms. After a 400 ms delay, a central fixation cross was displayed for 500 ms, followed by the presentation of the target stimulus for another 500 ms. Stimuli were presented on a light gray background. In order to minimize strategic adjustments in response speed across the incentive conditions, that is, more accurate but slower responding on gain and loss compared to no-incentive trials, we applied an adaptive response deadline. Depending on the proportion of time-out trials (*M* = 0.04, *SD* = 0.01), the response window was individually adjusted in steps of 100 ms within an overall range of 500–1500 ms (for a similar procedure, see Eppinger et al., 2009). After the key press, a blank screen was displayed for 500 ms and then visual feedback was provided for again 500 ms. If the participant failed to respond within the adaptive response time window, “Too Slow” feedback was shown. The next trial started after a randomly jittered 500 to 800 ms interval (see Figure 1 for a schematic overview of the trial procedure).

### Experimental procedure

The learning task consisted of a short practice block and 15 experimental blocks, with self-paced breaks every 30 trials. During the breaks, participants were presented with a feedback screen displaying the amount of money they had won so far (this value was always equal to or greater than zero, i.e., no negative scores were shown). Within one block, two stimuli were assigned to each incentive condition, yielding a total of six new stimuli per learning block. One of the two stimuli was mapped to the left response key and the other one to the right response key. Each stimulus was presented 10 times in pseudo-randomized order throughout the learning block, with the same stimulus appearing not more than two times in a row. The assignment of stimuli to incentive condition and response key was randomized across participants. The learning task took about 60 min to complete.

### Electrophysiological recording

The electroencephalogram (EEG) was recorded from 58 Ag/AgCl electrodes arranged according to the extended 10–20 system, referenced to the left mastoid, using Brain Amp DC Recorder (BrainVision recorder acquisition software). Data were sampled at 500 Hz in DC mode with a low-pass filter at 70 Hz. Impedances were kept below 5 kΩ. Electrodes placed on the outer canthi of the two eyes and on the infra- and supra-orbital ridges of the left eye recorded the horizontal and vertical electrooculograms. The data were re-referenced offline to the linked mastoids and band-pass filtered from 0.1 to 30 Hz. The impact of blinks and eye movements was corrected using an independent component analysis algorithm implemented in the BrainVision Analyzer Software Package (Brain products, Gilching, Germany). Trials containing EEG activity exceeding ±100 μV, changing more than 50 μV between samples or containing DC drifts were eliminated by a semiautomatic artifact inspection procedure.

### Data analyses

#### Behavioral data analyses

Responses exceeding the adaptive deadline were excluded from further analyses. To examine the course of learning, each block was split into five bins. The bins were created according to the number of stimulus repetitions, i.e., Bin 1 contained first and second presentation of the respective stimuli, Bin 2 third and fourth presentation, and so on. Within each bin, mean reaction times (RTs) and accuracy rates were computed for the three incentive conditions. The number of time-out trials did not differ (a) between incentive conditions, (b) across bins, or (c) after correct vs. erroneous responses in either age group (*p*s > 0.14). On average, 58 trials were included in each bin per condition.

#### ERP analyses

Artifact-free EEG data were segmented relative to response onset and baseline-corrected using the average voltage in a −200 to −50 ms preresponse interval. We defined the Ne as mean amplitude in a 0–50 ms time window following the response. The interval was chosen to capture the peak of the Ne in each age group (see **Figure 4**). As in previous studies (e.g., Hajcak et al., 2004; Wiswede et al., 2009), the Pe was computed as the mean amplitude in a 200- to 400-ms postresponse interval. Both, Ne and Pe were scored at the three midline electrodes FCz, Cz, and Pz, separately for correct and incorrect trials. In order to make sure that ERPs for correct and erroneous responses included the same number of epochs, we randomly selected a subsample of correct trials based on the individual error trial counts in each condition. Table 1 shows the mean number of EEG epochs that were used to quantify Ne and Pe in the three age groups.

**Table 1:** **Mean number (standard deviation) of EEG epochs that were included in the calculation of Ne and Pe**.

	**Children**	**Mid-Adolescents**	**Late adolescents**
Gain condition	69 (24)	45 (21)	57 (29)
Loss condition	70 (21)	45 (23)	60 (30)
No-incentive condition	74 (25)	50 (25)	68 (36)

#### Statistical analyses

Accuracy and ERP data were analyzed using repeated measures analyses of variance (ANOVAs). Whenever necessary, the Geisser-Greenhouse correction was applied (Geisser and Greenhouse, 1958) and corrected *p*-values are reported together with uncorrected degrees of freedom and the epsilon-values (ε). Planned comparisons were performed to decompose significant high-level interactions.

## Results

### Behavioral data

Reaction time and accuracy data were analyzed using ANOVAs with the between-subjects factor *Age group* (children, younger adolescents, older adolescents) and the within-subjects factors *Incentive condition* (gain, loss, and no-incentive) and *Bin* (Bins 1–5).

#### Reaction time

**Figure 2:**
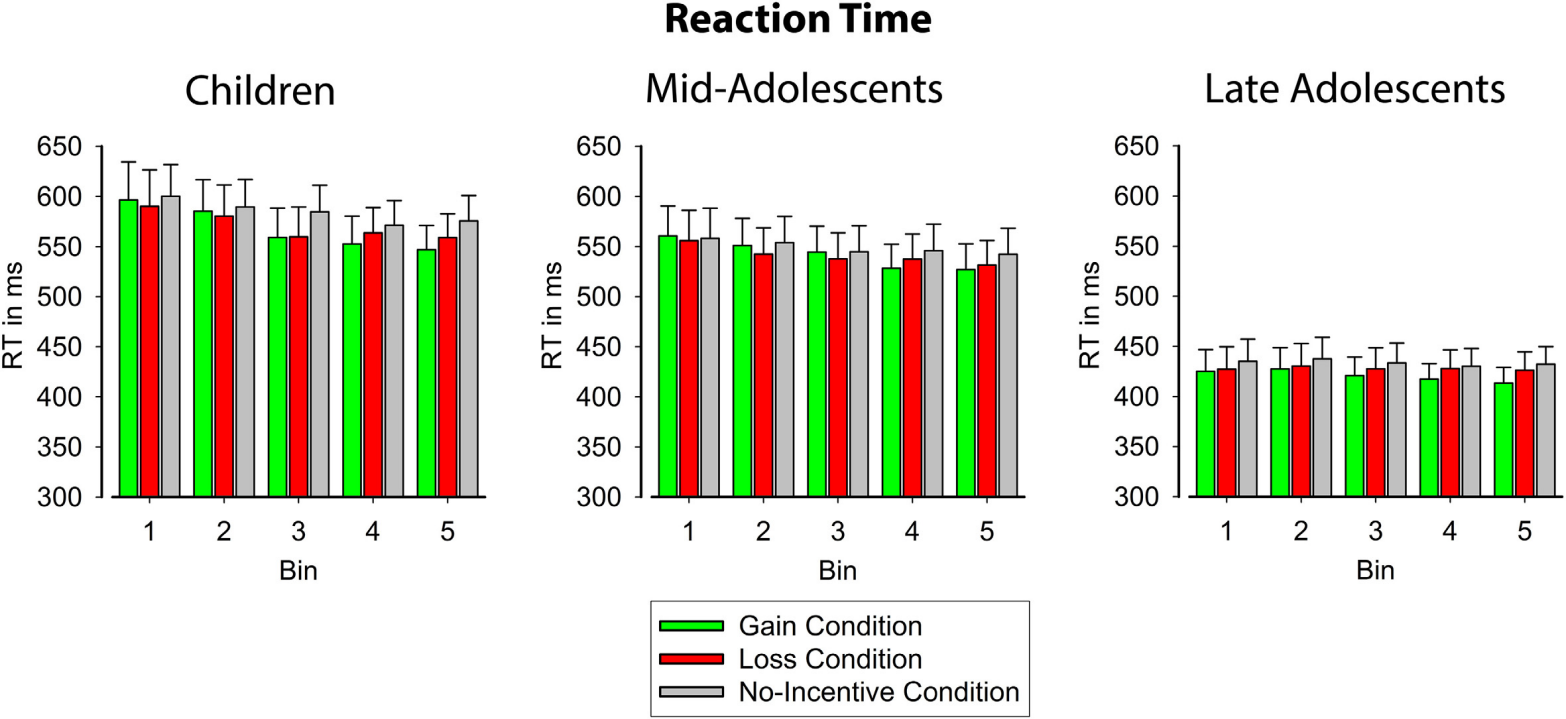
**Learning-related changes in reaction time for the three incentive conditions, displayed separately for children (left), younger adolescents (middle), and older adolescents (right)**. The x-axis shows the course of learning averaged into 5 bins. Error bars indicate standard error.

Figure 2 (see also Table 2) illustrates that RTs for all participants decreased with learning in each incentive condition [*F*_(4, 224)_ = 6.35, *p* < 0.01, ε = 0.38, η_*p*_^2^ = 0.10]. This was confirmed by a significant linear trend across bins [*F*_(1, 56)_ = 8.03, *p* < 0.01, η_*p*_^2^ = 0.13]. Moreover, RTs differed between the incentive conditions [*F*_(2, 112)_ = 6.44, *p* < 0.01, ε = 0.70, η_*p*_^2^ = 0.10] such that participants responded faster in gain and loss conditions compared to the no-incentive condition [*F*_(1, 56)_ = 7.37, *p* < 0.01, η_*p*_^2^ = 0.12]. As was indicated by an interaction of bin and incentive condition [*F*_(8, 448)_ = 3.35, *p* < 0.01, ε = 0.77, η_*p*_^2^ = 0.06], the learning-related speeding of responses varied for the three incentive conditions. Contrasts revealed that RTs decreased more rapidly in the gain compared to the loss condition [*F*_(1, 56)_ = 17.92, *p* < 0.001, η_*p*_^2^ = 0.24] as well as in gain and loss conditions compared to the no-incentive condition [*F*_(1, 56)_ = 4.57, *p* < 0.05, η_*p*_^2^ = 0.08]. There were also age differences in overall RT across age groups [*F*_(2, 56)_ = 10.23, *p* < 0.001, η_*p*_^2^ = 0.27]. Older adolescents responded faster than both younger adolescents and children [*F*_(1, 56)_ = 19.80, *p* < 0.001, η_*p*_^2^ = 0.26], whereas response latencies did not differ between the latter two age groups [*F* < 1, *p* = 0.38].

**Table 2:** **Mean reactions times (standard deviations) in ms for each condition and bin of the learning task**.

**Condition**	**Bin**	**Children**	**Mid-Adolescents**	**Late adolescents**
Gain	1	597 (165)	561 (134)	425 (98)
	2	585 (137)	551 (122)	427 (95)
	3	559 (127)	544 (116)	421 (83)
	4	553 (120)	528 (106)	417 (68)
	5	547 (105)	527 (115)	413 (70)
Loss	1	590 (158)	546 (136)	427 (100)
	2	580 (135)	542 (118)	430 (100)
	3	560 (129)	538 (116)	427 (94)
	4	564 (110)	538 (111)	428 (84)
	5	559 (103)	532 (108)	426 (82)
No-incentive	1	600 (137)	558 (135)	435 (99)
	2	590 (119)	554 (117)	438 (97)
	3	585 (115)	545 (116)	434 (89)
	4	571 (106)	546 (119)	430 (80)
	5	576 (110)	542 (116)	432 (78)

#### Accuracy

**Figure 3:**
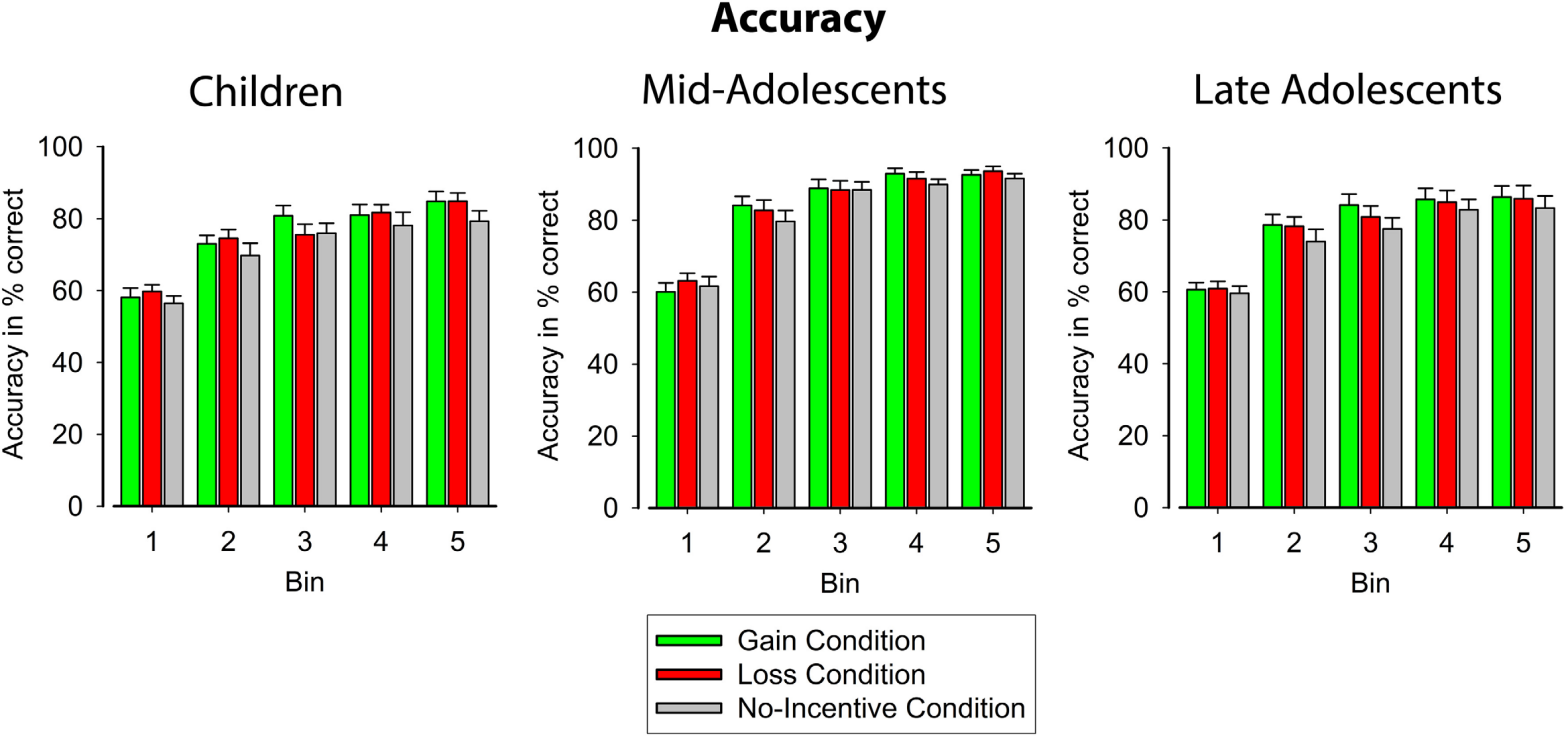
**Learning-related changes in accuracy rates for the three incentive conditions, displayed separately for children (left), younger adolescents (middle), and older adolescents (right)**. The x-axis shows the course of learning averaged into 5 bins. Error bars indicate standard error.

Accuracy learning curves for the three age groups in the three incentive conditions are shown in Figure 3 and Table 3. Accuracy increased with age [*F*_(2, 56)_ = 4.30, *p* < 0.05, η_*p*_^2^ = 0.13] such that younger and older adolescents showed higher overall accuracy than children [*F*_(1, 56)_ = 5.16, *p* < 0.05, η_*p*_^2^ = 0.08] but did not significantly differ from each other (*p* = 0.07, η_*p*_^2^ = 0.05). As expected, all participants became more accurate across learning blocks [*F*_(4, 224)_ = 302.389, *p* < 0.001, ε = 0.38, η_*p*_^2^ = 0.84]. The course of learning, however, differed for the three age groups [*F*_(8, 224)_ = 2.60, *p* < 0.05, ε = 0.59, η_*p*_^2^ = 0.09]. Contrasts revealed stronger quadratic [*F*_(1, 56)_ = 46.49, *p* < 0.001, η_*p*_^2^ = 0.45] and cubic trends [*F*_(1, 56)_ = 12.86, *p* < 0.01, η_*p*_^2^ = 0.18] across bins in younger and older adolescents than in children, indicating that older participants learned faster and reached asymptote levels of accuracy earlier, while children's performance continued to improve more steadily throughout the learning blocks. Importantly, accuracy varied across the incentive condition [*F*_(2, 112)_ = 10.56, *p* < 0.001, ε = 0.86, η_*p*_^2^ = 0.16]. Participants showed better performance in gain and loss conditions compared to the no-incentive condition [*F*_(1, 56)_ = 15.52, *p* < 0.001, η_*p*_^2^ = 0.22], while accuracy scores did not differ reliably between the former two conditions (*F* < 1, *p* = 0.55). Similar to the RT data, however, there were no significant age differences in the influence of incentive-value on learning performance.

**Table 3:** **Mean accuracy rates (standard deviations) for each condition and bin of the learning task**.

**Condition**	**Bin**	**Children**	**Mid-adolescents**	**Late adolescents**
Gain	1	0.58 (0.11)	0.60 (0.11)	0.61 (0.09)
	2	0.73 (0.10)	0.84 (0.11)	0.79 (0.13)
	3	0.81 (0.12)	0.89 (0.11)	0.84 (0.13)
	4	0.81 (0.13)	0.93 (0.07)	0.86 (0.14)
	5	0.84 (0.12)	0.93 (0.06)	0.86 (0.14)
Loss	1	0.60 (0.08)	0.63 (0.09)	0.61 (0.09)
	2	0.75 (0.11)	0.83 (0.13)	0.78 (0.12)
	3	0.76 (0.13)	0.89 (0.12)	0.81 (0.14)
	4	0.82 (0.10)	0.92 (0.08)	0.85 (0.14)
	5	0.85 (0.10)	0.94 (0.06)	0.86 (0.16)
No-incentive	1	0.56 (0.09)	0.62 (0.12)	0.60 (0.09)
	2	0.70 (0.15)	0.80 (0.14)	0.74 (0.15)
	3	0.76 (0.12)	0.88 (0.10)	0.78 (0.14)
	4	0.79 (0.16)	0.90 (0.08)	0.83 (0.13)
	5	0.79 (0.13)	0.92 (0.07)	0.83 (0.15)

### Electrophysiological data

**Figure 4:**
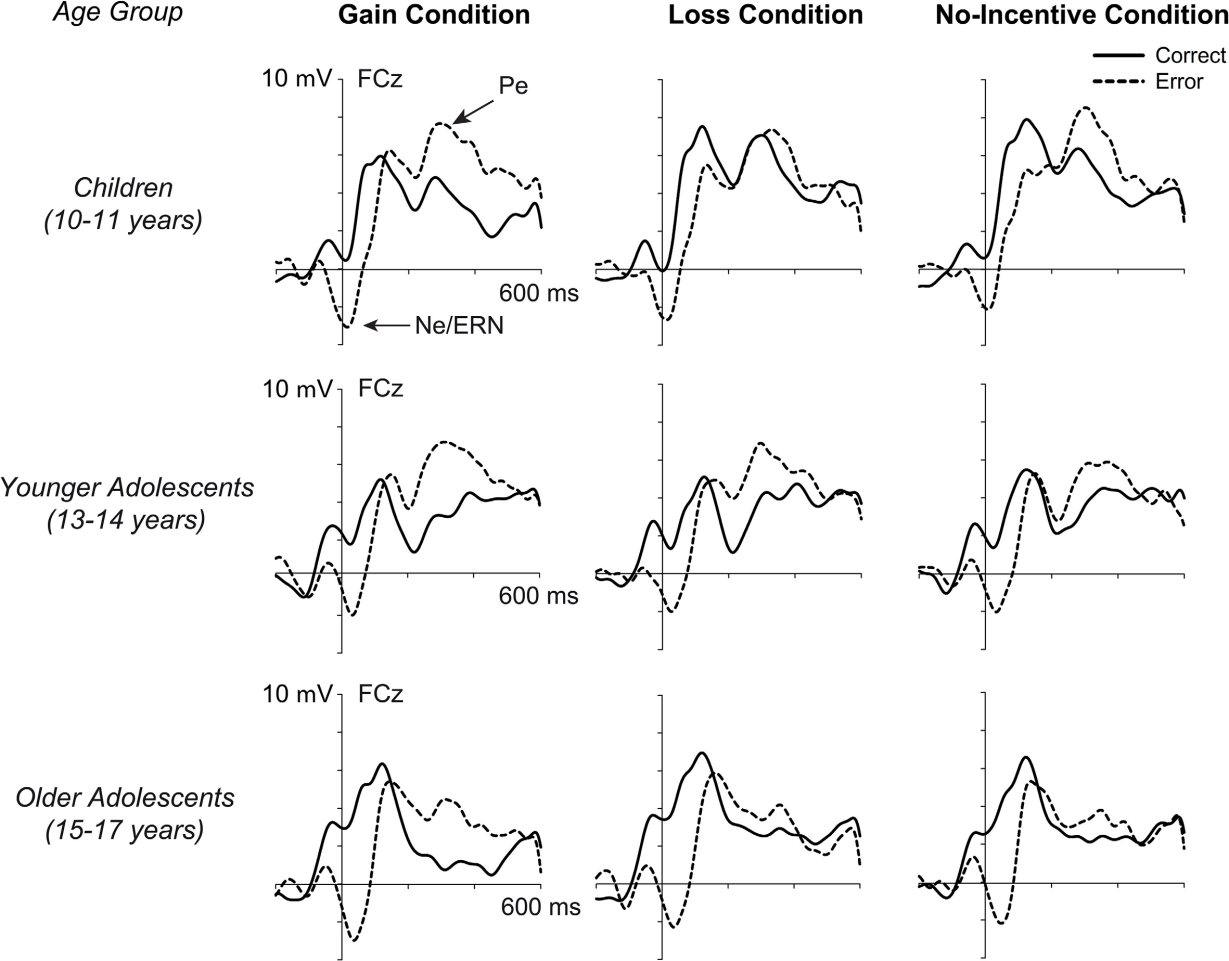
**Response-locked grand average ERPs for the three incentive conditions, displayed separately for correct responses (solid lines) and incorrect responses (dashed lines), for children (top), younger adolescents (middle), and older adolescents (bottom) at electrode FCz**.

Figure 4 shows the ERPs to correct and erroneous responses at electrode site FCz, separately for the three incentive conditions for children, younger adolescents, and older adolescents. In all age groups, the Ne is evident as a fronto-centrally distributed negative deflection that is larger after erroneous than correct responses. The Ne increases with age, but is not clearly modulated by incentive condition. Following the Ne, the Pe can be observed as a positive deflection. In contrast to the Ne, the Pe seems to be smaller in older adolescents than in the two younger age groups and varies across the incentive conditions. Specifically, the Pe appears to be larger in the gain condition compared to loss and no-incentive condition.

Ne and Pe were subjected to separate ANOVAs involving the between-group factor *Age group* (children, younger adolescents, older adolescents) and the within-subjects factors *Incentive condition* (gain, loss, and no-incentive) and *Correctness* (correct vs. incorrect). In order to control for influences of RT differences between groups, we additionally ran ANVOVAs including mean response latencies as covariate. These analyses did not yield evidence that the ERP findings varied as a function of RT.

#### Error negativity

Analyses confirmed that the Ne was larger on incorrect compared to correct trials [*F*_(1, 56)_ = 77.99, *p* < 0.001, η_*p*_^2^ = 0.58] and increased from posterior to anterior sites [correctness × electrode: [*F*_(2, 112)_ = 28.48, *p* < 0.001, ε = 0.70, η_*p*_^2^ = 0.34]. As was indicated by a significant interaction of age group and correctness [*F*_(2, 56)_ = 6.66, *p* < 0.01, η_*p*_^2^ = 0.19], the Ne increased with age. Contrasts revealed that the amplitude difference between correct and erroneous trials was larger in the two adolescent groups compared to children [*F*_(1, 56)_ = 5.73, *p* < 0.05, η_*p*_^2^ = 0.09] as well as in 15–17-year-olds compared to 13–14-year olds [*F*_(1, 56)_ = 12.52, *p* < 0.01, η_*p*_^2^ = 0.18]. However, we found no evidence that the Ne reliably varied as a function of incentive condition in either age group (*p*s > 0.27).

#### Error positivity

As expected, there was a larger positivity on erroneous compared to correct trials [*F*_(1, 56)_ = 39.08, *p* < 0.001, η_*p*_^2^ = 0.41] and this amplitude difference was more pronounced at posterior than anterior scalp sites [correctness × electrode: *F*_(2, 112)_ = 9.62, *p* < 0.01, ε = 0.67, η_*p*_^2^ = 0.18]. Moreover, we found age-related differences in Pe amplitude [age group x correctness: *F*_(2, 56)_ = 3.39, *p* < 0.05, η_*p*_^2^ = 0.11]. Contrasts showed that the Pe was reduced in 15–17-year olds compared to the two younger age groups [*F*_(1, 56)_ = 6.50, *p* < 0.05, η_*p*_^2^ = 0.10], but did not significantly differ in 13–14-year-olds and 10–11-year-olds (*p* = 0.63). Most importantly, Pe amplitude differed between incentive conditions [*F*_(2, 112)_ = 9.62, *p* < 0.001, ε = 0.89, η_*p*_^2^ = 0.15], such that it was enhanced on gain trials compared to loss and no-incentive trials [*F*_(1, 56)_ = 9.98, *p* < 0.01, η_*p*_^2^ = 0.15]. This effect, however, did not significantly vary as a function of age (*p* = 0.38).

## Discussion

In this study, we examined developmental differences in motivational influences on error processing—as reflected in Ne and Pe—and error-induced learning, comparing children (10–11 years), mid-adolescents (13–14 years) and late adolescents (15–17 years). We used an incentivized reinforcement learning task, in which errors resulted in losing money (loss condition), failure to gain money (gain condition), or neither (no-incentive condition).

Behaviorally, participants in all age groups responded more quickly and accurately to stimuli in gain and loss conditions than to those in the no-incentive condition. Thus, even 10–11-year-old children were able to efficiently use motivational cues in order to maximize outcomes of their task performance. The behavioral data, however, did not support the idea that motivational salience has a greater impact on learning-related performance adjustment in adolescents than in children. Instead, the current findings are in line with results from previous studies using “non-affective” decision-making paradigms that reported linear age-related performance improvements (e.g., Crone et al., 2008; Van Duijvenvoorde et al., 2008; Van den Bos et al., 2012). Similarly, Van der Schaaf et al. (2011) found that overall accuracy in a reversal-learning task (including reversal and non-reversal trials) increased with age, reaching asymptote at adolescence. However, the authors observed an inverted U-shaped relationship between age and performance on reversal trials, peaking at adolescence. Benefits of adolescents' aberrant sensitivity to salient motivational cues hence might be limited to situations that require a particularly high degree of flexibility, such as the need for rapid behavioral reversal in volatile environments. Thus, one reason for the failure to obtain non-linear age-related changes in the present study might have been that we used a deterministic learning task with fixed stimulus-response mappings, causing a predictable and stable environment.

In line with findings from previous developmental studies on performance monitoring (e.g., Davies et al., 2004; Santesso et al., 2006; Ladouceur et al., 2007), the Ne increased with age until late adolescence. The reduction of Ne amplitude in younger participants has been linked to the protracted structural and functional development of the medial prefrontal performance monitoring system (cf. Ladouceur et al., 2007; Torpey et al., 2009), especially the ACC (the putative neural source of the Ne; Gehring et al., 2012). Importantly, there is evidence to suggest that age difference in the Ne may be attributed to children's deficits in task performance rather than developmental changes in the neural structures underlying performance monitoring (e.g., Eppinger et al., 2009). In the present study, however, we observed larger Ne amplitudes in older compared to younger adolescents in the absence of significant differences in overall accuracy. The current findings thus corroborate the view that neural systems underlying the Ne continue to develop throughout adolescence (Ladouceur et al., 2007).

This view would also be consistent with our observation that the Ne was not modulated by the incentive manipulation in children and adolescents, whereas previous studies demonstrated that the Ne is sensitive to such motivational influences in adults (e.g., Hajcak et al., 2005; Potts, 2011). Notably, we recently tested a sample of young adults using a highly similar reinforcement learning paradigm that included probabilistic instead of deterministic stimulus-response mappings and found a larger Ne in the loss condition compared to both gain and no-incentive conditions (Unger and Kray, unpublished data). The present results parallel findings by Torpey et al. (2009) in younger children (5–7 years), but contrast with the study by Kim et al. (2005), in which the presence of a peer during task performance was associated with an increase in the Ne in 7–8-year-olds. The latter finding raises the more general question of whether monetary incentives are sufficiently salient motivational cues for children and adolescents. Although the present data show incentive-related improvements in task performance, there is evidence to support the notion that social-affective incentives (e.g., peer admiration) may play a more prominent role in adolescence (Crone and Dahl, 2012). Despite the plausibility of this hypothesis, previous work demonstrated that monetary gains and losses have a substantial impact on decision making in adolescents (e.g., Van Duijvenvoorde et al., 2010).

While the Ne did not vary as a function of error-value, both children and adolescents showed a larger Pe to errors in the gain condition compared to loss and no-incentive conditions. This is in contrast to results from adult studies, in which the Ne but not the Pe was enhanced for high-value errors (e.g., Hajcak et al., 2005; Potts, 2011). Given that these two components are thought to reflect functionally dissociable mechanisms (Overbeek et al., 2005; Ridderinkhof et al., 2009; Steinhauser and Yeung, 2010), the present findings indicate that motivational influences on error processing qualitatively change across development. Interestingly, recent work established a specific link between error detection mechanisms and the Pe, whereas the Ne might be related to more general aspects of performance monitoring—such as conflict detection or tracking of error/reward likelihood—rather than to error processing itself (Steinhauser and Yeung, 2010). Specifically, Steinhauser and Yeung (2010) suggested that the Pe reflects a process that feeds available evidence for the decision that a response error has occurred into an internal error detection system and hence may support deliberate performance adjustments. According to this view, the current findings indicate that participants were more certain about error commission, i.e., had stronger evidence that an error occurred—in the gain compared to loss and no-incentive conditions. While conscious error detection may have contributed to performance optimization on gain trials, it remains to be determined which mechanisms underlie improved performance in the loss condition. One possibility is that motivationally salient loss feedback is more robustly maintained in working memory (Frank et al., 2007).

Moreover, the inverse age-related changes in Pe (decrease) and Ne (increase) raise the interesting possibility that error-related remedial behaviors might rely on different mechanisms across development. While larger Pe amplitudes in children and mid-adolescents may reflect that younger participants gather more evidence to support conscious error detection (Steinhauser and Yeung, 2010), the enhanced Ne in older adolescents indicates stronger recruitment of more general performance monitoring mechanisms (e.g., conflict monitoring).

Some limitations of the current study should be noted. First, the learning paradigm might have been insensitive to unique features of motivational processing in adolescence. Clearly, future studies are needed to test whether subcortical mechanisms can exert beneficial influences on adolescents' performance in salient social-affective contexts or situations that require higher behavioral flexibility (e.g., volatile and uncertain environments). Second, one could argue that participants in the youngest age group were too close to adolescence and hence did not provide an appropriate “baseline.” However, other studies that did find unique effects of motivational-affective variables on adolescents' decision making covered a similar age range (cf. Somerville et al., 2010). Moreover, sensitivity to incentives has been shown to peak between ages 14 and 16 (Somerville and Casey, 2010). Thus, it seems unlikely that limitations of age range account for the failure to obtain age differences in incentivized learning. Nonetheless, it is important to mention that age does not provide a precise measurement of pubertal development. Future studies thus should include a direct assessment of pubertal status.

Notably, comparisons of the present data with previous findings in adults suggest that (1) the medial prefrontal performance monitoring system underlying the Ne undergoes functional change until late adolescence and (2) incentive-related modulations in performance monitoring are reflected in the activity of at least partially dissociable neural systems in children and adolescents (modulations in the Pe) vs. young adults (modulations in the Ne) that may support more deliberate vs. automatic, preconscious forms of performance adjustment, respectively. Since our study did not include an adult group, these conclusions need to be tested by future studies applying the same learning paradigm in children, adolescents and young adults. Furthermore, future research may probe whether motivational factors influence the relationship between neural error signals and error-related performance adjustment on a single-trial basis.

To conclude, the present findings do not support the idea that incentives generally have a stronger impact on feedback-based learning in early and late adolescence than in late childhood. Instead, the behavioral data showed that both children and adolescents efficiently used incentive cues to optimize performance outcomes, with no systematic differences between salient reward (gain condition) and salient punishment (loss condition). However, the ERP data suggested that gain but not loss anticipation is associated with enhanced recruitment of error processing mechanisms as reflected in the Pe that are thought to support conscious error detection and deliberative performance adjustment.

### Conflict of interest statement

The authors declare that the research was conducted in the absence of any commercial or financial relationships that could be construed as a potential conflict of interest.
